# Prevalence of Missed Injuries in Multiple Trauma Patients at a Level-1 Trauma Center in Saudi Arabia

**DOI:** 10.7759/cureus.34805

**Published:** 2023-02-09

**Authors:** Ibrahim Al Babtain, Yara Almalki, Deemah Asiri, Nazish Masud

**Affiliations:** 1 General Surgery, King Abdulaziz Medical City, Riyadh, SAU; 2 College of Medicine, King Saud Bin Abdulaziz University for Health Sciences, Riyadh, SAU; 3 Department of Biostatistics, Epidemiology and Environmental Health Sciences, Georgia Southern University, Statesboro, USA

**Keywords:** glasgow coma scale score, multiple trauma, saudi arabia, delayed diagnosis, missed injury

## Abstract

Background

Missed injuries are defined as injuries neither detected in the emergency department (ED) nor after admission to the hospital. The objective of this research was to identify missed injury rates, contributing factors, and clinical outcomes.

Methods

A total of 657 trauma patients’ records were retrospectively reviewed after admission to King Abdulaziz Medical City (KAMC) during the period from January 2016 to December 2018. Patients’ demographic characteristics, presence of a missed injury, and Glasgow Coma Scale (GCS), Revised Trauma Score (RTS), and Injury Severity Score (ISS) were assessed.

Results

Among 657 patients who were admitted to our emergency department, only 11 (1.7%) patients were reported to have a missed injury during the hospital stay. None of those missed injuries contributed to the overall mortality. Higher GCS is a protective factor for missed injury with OR=0.12-0.81 and p-value=0.01. RTS and intensive care unit (ICU) stays were borderline although p-value=0.05 and OR=9 for RTS. Both longer ICU stays and high RTS were related to a higher risk of missed injury.

Conclusion

In our study, the prevalence of missed injuries was on the lower end of the spectrum in comparison to multiple published data. The most common missed injuries were fractures and joint dislocations of extremities. None of those missed injuries were life-threatening or contributed to overall mortality. Higher GCS was a protective factor against missed injuries while high RTS and longer ICU stays were related to a higher likelihood of developing missed injuries during the hospital course.

## Introduction

A missed injury is defined as an injury that was not detected or diagnosed in the emergency department (ED) or after hospital admission [1-3]. Missed injuries are preventable and have multiple implications for patients and healthcare workers. Those injuries result in a late diagnosis and, consequently, a delay in providing treatment, which may lead to prolonged hospitalization, increased cost, and, rarely, increased morbidity and mortality rates [4,5].

During the evaluation of trauma patients in the ED, missed injuries may occur. The initial approach for these patients should follow the guidelines of the Advanced Trauma Life Support (ATLS) by the American College of Surgeons, which consists of two surveys for trauma patients. The primary survey is established to identify life-threatening injuries immediately in the first minutes of ED arrival and treat those injuries immediately. After stabilizing patients, the secondary survey uses a head-to-toe approach to detect all other (major and minor) injuries [6].

Several risk factors have been defined in previous literature that may result in a delayed diagnosis of the injury. Higher Injury Severity Score (ISS), altered level of consciousness, lower Glasgow Coma Scale (GCS), intubation, need for intensive care, presence of polytrauma, injury as a consequence of an automobile accident, longer length of intensive care unit (ICU) admission, and emergency surgery have been reported to be factors associated with missed injuries [7-13]. Missed injuries can be found in the upper and lower extremities and have also been reported in the head and abdomen [2,7,10,13].

The reported incidence of missed injury rates varies from 0.6% to 39% [14]. These differences were a result of different research methods and criteria implemented to detect missed injuries. Among local studies that were conducted in Saudi Arabia, a 1996 study (n= 638) was done in the orthopedic department to determine the incidence rate of musculoskeletal injuries. At King Khalid University Hospital, 80 missed musculoskeletal injuries in 50 patients were analyzed. The reported incidence rate after the orthopedic department admission was 6% of the patients. The most common sites of missed injury were the knee (26 injuries), the foot and ankle (14 injuries), and the hip and pelvis (13 injuries) [15]. In 1996, there was no appropriate trauma team activation, and there was no team for the assessment of trauma patients.

Currently, there is no recent research that has been conducted to assess the incidence of missed injuries in Saudi Arabia in King Abdulaziz Medical City (KAMC), the only tertiary hospital that is a level-1 trauma center in Riyadh, with regard to trauma team activation. The main goal of this research is to determine missed injuries and identify the contributing factors and clinical outcomes of missed injuries.

## Materials and methods

Study design and settings

This was a retrospective cohort study conducted on trauma patients after trauma team activation to determine the incidence of missed injuries. This study was conducted in King Abdulaziz Medical City (KAMC) in Riyadh, which is a level-1 trauma center. In KAMC, it was estimated that more than 1100 trauma patients were admitted to trauma centers annually.

This research was reviewed and approved by the institutional review board at King Abdullah Medical Research Center.

Participants

All trauma patients, approximately 2200, who were admitted to KAMC after trauma team activation between January 2016 and December 2018 were included in this study retrospectively. In total, 657 trauma patients’ charts were reviewed after admission to KAMC and trauma team activation within that time period. Primary and secondary surveys, whole-body CT scans for trauma, extended Focused Assessment with Sonography in Trauma (e-FAST), and tertiary surveys were all conducted in accordance with the trauma team algorithm. Patients were included in this study if they were above 15 years old, Saudi or non-Saudi, and those who died before undergoing tertiary survey or transferred from another hospital within the first 24 hours of the trauma. Patients were excluded if they expired in ED.

Data sources

Two trained medical students supervised by the principal investigator extracted demographic and injury-related data from the electronic records of all cases. Electronic Best-Care chart data collection by the students was reviewed by the principal investigator for accuracy. The people collecting all the data were uninvolved in treating the patients. The following parameters were collected from the patient’s medical records: demographic-related factors included age, gender, nationality, and intubation status, while injury-related data included the number, type, and mechanisms of injury. GCS is utilized to assess the motor, verbal, and eye responses, and the total score has values between three, being the lowest and showing a poorer prognosis, and 15 being the highest to categorize the level of consciousness [16]. Revised Trauma Score (RTS) is a scoring system used initially to estimate the severity of the trauma and consists of the GCS, systolic blood pressure, and respiratory rate. ISS uses the worst injury score in three separate body regions in which the lower the sum score, the more severe the injury. Inadequate clinical examination and patients who needed to be intubated were contributing factors to delayed diagnosis and were calculated by one co-investigator [17,18]. Circumstantial variables included time of arrival to the ED, time of diagnosis, duration until the diagnosis of injuries, and the length of hospital stay. In addition, all radiological studies, emergent operations, and hospital mortality within the first 24 hours were recorded. Treatment and hospital mortality after 24 hours were also recorded.

Statistical analysis

All retrieved data was stored in a password-protected computer in the Principal Investigator’s office. An extraction sheet was used, and data were entered in Microsoft Excel version 16.9 (Microsoft Corporation, Redmond, WA) to conduct data checking, proofing, and cleaning. Statistical analysis was performed using Statistical Package for the Social Sciences version 22 (IBM Corp., Armonk, NY). Descriptive statistics were used to demonstrate patients' demographic characteristics, in which frequencies and percentages were used to describe categorical variables, such as gender, mechanisms of injury, and intubation status, while mean and standard deviation were used for continuous variables such as the patient's age and number of missed injuries. Also, descriptive statistics were used to compute GCS, RTS, and ISS total scores. T-test and analysis of variance (ANOVA) were used for continuous variables to compare demographic groups. Inferential statistics (chi-square test, p <0.05) were applied to assess the significance among categorical variables. The p-value was set at 0.05 for all the tests applied.

Ethical approval 

Ethical approval of this study was obtained from the IRB (RC20/062/R) on September 16, 2021. No medical record numbers were obtained to assure confidentiality, and informed consent was not required.

## Results

Patient characteristics

A total of 657 patients' charts were reviewed with a mean age of 31±12 years with a majority (611; 93%) of patients being males. The most frequent mechanism of injury was motor vehicle accidents (MVAs) representing 382 (58%) followed by pedestrian accidents (94; 14%). Four-hundred thirty-nine (439; 67%) patients were intubated with the most frequent cause of intubation being traumatic brain injury (TBI) represented by loss of consciousness (LOC) in 314 (48%) with a mean±SD of 5±6 days. Two-hundred ninety-five (295; 45%) patients were shifted to the ICU with an average of 14±15 days, while 210 (32%) were transferred immediately to the operating room. One hundred (100; 15%) patients expired after their admission to the hospital. RTS had a mean±SD of 10±2 while ISS had a mean±SD of 19±12. GCS had an average of 11±4, revealing that most of the study population had moderate GCS scores. On average, hospital length of stay had a mean±SD of 29±40. The number of injuries had a mean±SD of 4±2 with head and neck injuries being 476 (73%) followed by thoracic injuries reported as 341 (52%) injuries (Table 1).

**Table 1:** Patient Characteristics (n=657) * Fisher's exact statistic significant at 0.05 ER: emergency room; ICU: intensive care unit; LOC: loss of consciousness; OR: operating room; SCI: spinal cord injury; TBI: traumatic brain injury

Variables	Category	The presence of missed Injury	P-value
All Patient	Absent (N=646)	Present (N=11)
N	%	N	%	N	%
Gender	Male	611	93%	600	93%	11	100%	1.0
Female	46	7%	46	7%	0	Nil
Nationality	Non-Saudi	248	38%	246	38%	2	18%	0.22
Saudi	409	62%	400	62%	9	82%
Mechanism of injury	Motor Vehicle Accident	382	58%	375	58%	7	64%	0.7
Motorcycle Accident	41	6%	40	6%	1	9%
Pedestrian	94	14%	92	14%	2	18%
Homicide and Injury Purposely Inflicted by Other Persons	57	9%	57	9%	0	Nil
Suicide and Self-Inflicted Injury	7	1%	7	1%	0	Nil
Burn due to Fire or Flames	25	4%	25	4%	0	Nil
Falls	20	3%	19	3%	1	9%
Other Accidents	31	5%	31	5%	0	Nil
Intubation status	No	218	33%	214	33%	4	36%	0.75
Yes	439	67%	432	67%	7	64%
Causes of intubation	Not intubated	242	37%	238	37%	4	36%	0.73
TBI\LOC	314	48%	307	48%	7	64%
Chest Injury=2	21	3%	21	3%	0	Nil
Intubation in OR	3	1%	3	1%	0	Nil
SCI	3	1%	3	1%	0	Nil
Other	74	11%	74	12%	0	Nil
ER. Disposition	Ward	124	19%	121	19%	3	27%	0.67
ICU	295	45%	289	45%	6	55%
OR	210	32%	208	32%	2	18%
Morgue	19	3%	19	3%	0	Nil
Burn Unit	9	1%	9	1%	0	Nil
Living Status	Alive	557	85%	546	85%	11	100%	0.38
Dead	100	15%	100	16%	0	Nil
Type of injury	Blunt	562	86%	551	98%	11	2%	1.00
Penetration (Stab)	31	5%	31	100%	0	Nil
Penetration (Gunshot)	35	5%	35	100%	0	Nil
Penetration (Other)	2	Nil	2	100%	0	Nil
Burn/Scald	27	4%	27	100%	0	Nil

Prevalence of missed injury

**Figure 1:**
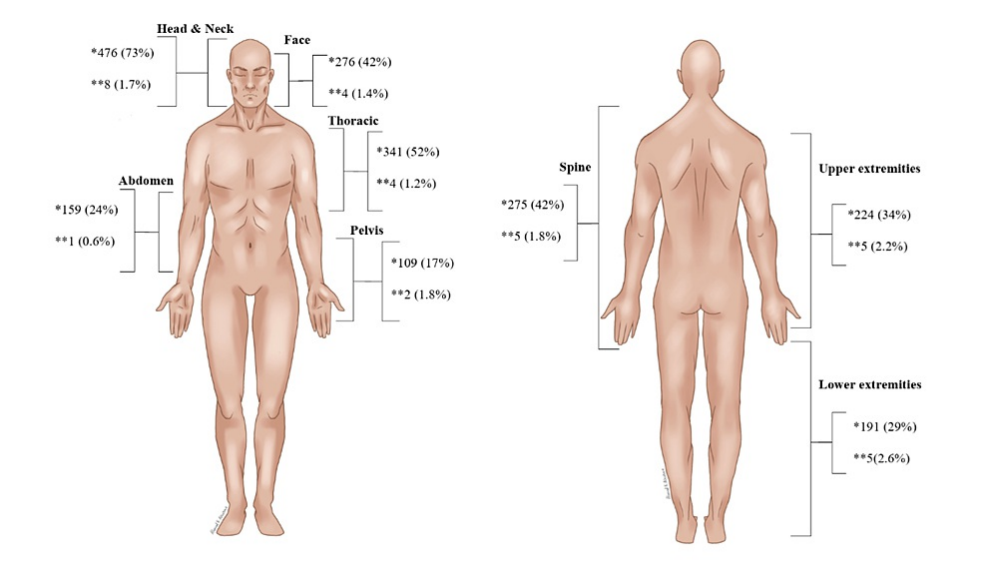
Summary of anatomical regions of injuries among trauma patients * Injury among all trauma patients (n=657) ** Injury among patients with missed injury (n=11) *** Figure drawn by Dr. Alanoud Alsubaie

Among 657 patients who were admitted to our ED, only 11 (1.7%) patients were reported to have a missed injury during the hospital stay. All of those patients are male. The predominant mechanism of missed injury was MVAs representing seven (64%) injuries. Seven (64%) patients were intubated with TBI as the main cause of intubation. Six (55%) patients were transferred to the ICU, three (27%) patients were shifted to the ward, and only two (18%) patients were transferred immediately to the operating room. All patients with missed injuries that were diagnosed during the hospital course were alive. Out of the 476 who had a head injury, eight (1.7%) had missed injuries detected later during the hospital admission, followed by upper extremities in five (2.2%) patients, and lower extremities involving five (2.6%) patients. Those missed injuries were eight (72.7%) fractures in the peripheral extremity regions, followed by two (18.1%) joint dislocations/displacements, and one (9.1%) patient with brain hematoma (Figure 1).

Predictors of missed injury

Multiple variables were included in logistic regression modeling such as age, GCS, RTS, ISS, hospital length of stay, emergency room (ER) length of stay, ICU days, and intubation days. The GCS is significantly associated with missed injury, in which higher GCS is a protective factor for missed injury with (OR=0.31, 95% CI: 0.12-0.81, p-value=0.01). RTS and ICU length of stay are borderline. High chances of missed injury are associated with higher RTS values (OR=9, p=0.05). Additionally, there was a higher likelihood of missed injury with longer ICU stays (Table 2).

**Table 2:** Predictors of missed injuries among multiple trauma patients * The chi-square/ Fisher exact test as applicable significant at <0.05 ER: emergency room; GCS: Glasgow Coma Scale; ICU: intensive care unit; ISS: injury severity score; RTS: revised trauma score

Variables	B	Odds ratio	95% C.I.	P-value
Lower	Upper	
Age	0.041	1.042	0.989	1.097	0.120
GCS	-1.155	0.315	0.122	0.817	0.018
RTS	2.241	9.403	0.928	95.218	0.058
ISS	-0.015	0.985	0.904	1.073	0.726
Hospital length of stay (Days)	-0.017	0.983	0.955	1.011	0.223
ER length of stay (Hours)	0.165	1.18	0.966	1.44	0.104
ICU admission (Days)	0.088	1.092	0.998	1.193	0.055
Intubation (Days)	-0.058	0.944	0.783	1.137	0.541

## Discussion

A missed injury is defined as an injury that was not detected or diagnosed in the ED or after ICU admission [19]. Missed injuries are preventable and have multiple implications for patients and healthcare workers. The delayed detection of injuries and, in turn, delay in providing treatment may lead to prolonged hospitalization, increased cost, and, though rarely, increased morbidity and mortality rates [20,21]. This study aimed to determine the prevalence of missed injuries among trauma patients. A total of 657 patients, with an age range of 19-43 and an average age of 31, were included in this study. Six-hundred eleven (611; 93%) patients were males due to this research being conducted in a level-1 trauma center during a period when women were not legally allowed to drive. Three-hundred eighty-two (382; 58%) were MVAs - being the predominant mechanism of injury, while 94 (14%) were pedestrian accidents. This can be explained as MVAs account for 32.3% of all adult mortality in Saudi Arabia despite preventive measures and programs, potentially making it the second leading cause of death in the country. In Saudi Arabia, MVAs result in more than 19 deaths each day and almost four injuries every hour [22,23].

Prevalence of missed injuries in Saudi Arabia

Among 685 patients who were admitted to our emergency department, only 11 (1.7%) patients were reported to have a missed injury during the hospital course. Multiple studies had prevalence rates of missed injuries ranging from 0.6% to 39% [1-4,13,14,24,25]. In more recent studies, such as the study by Lawson et al., the incidence rate of delayed diagnosis was 90 (0.34%) of 26,264 patients, which is lower than in our study [24]. In Lee WC et al., the overall incidence of delayed diagnosis of injury was 12.1% of a total of 976 trauma patients admitted to surgical ICU [26]. The study by Tammelin et al. had a similar prevalence of 2.6% missed injuries among all injuries diagnosed in the hospital and within three months after trauma [13]. Our study prevalence rate is similar to multiple studies although these differences were a result of different research methods and criteria implemented to detect missed injuries. Our missed injury rate of 1.7% is on the lower end of the spectrum in comparison to published data. In our study, all those patients are males. The most frequent mechanism causing missed injury was MVAs representing seven (64%) missed injuries. In most studies, patients with missed injuries were reported to be mostly males and MVA victims [4,13,25]. In our study, six (55%) patients were transferred to the ICU, three (27%) patients were shifted to the ward, and only two (18%) patients were transferred immediately to the operating room. All patients with missed injuries that were detected during the hospital course were alive. It is worth mentioning that these missed injuries did not result in mortality in our study. Similar findings in multiple other studies have shown that none of the delayed diagnoses contributed to the overall morbidity and mortality of the patients [5,10,11,13,19,27]. Out of the 476 patients who had a head injury or TBI, eight (1.7%) patients had a missed injury detected later during the hospital course. In Lee WC et al., patients who sustained injuries in the head and abdomen were at a higher risk to have a delayed diagnosis of injury among patients admitted to the surgical ICU [26]. Eleven patients had a missed injury detected later in the hospital course. One (9%) patient had a brain hematoma, and 10 (91%) patients had reported fractures and dislocations of upper and lower extremities. It can be explained through the current literature that missed injuries typically involve upper and lower extremity fractures. In Fitschen-Oestern et al., 144 (6.5%) of 2532 patients had missed foot injuries [28]. Similarly, in Fitschen-Oestern et al., among 10971 patients with hand injuries, 727 (6.6%) patients had missed hand injuries [29]. In Wei C-J et al., missed fractures in the extremities occurred at a rate of 3.7% with 115 fractures in 108 patients being missed in the initial radiological reports [30].

Predictors of missed injuries

Multiple variables were studied in logistic regression modeling such as age, GCS, RTS, ISS, hospital length of stay, ER length of stay, ICU length of stay, and intubation in days. In our study, high GCS was significantly associated with missed injuries as a protective factor during a hospital stay. Many studies showed that lower GCS is a negative predictor and statistically significant for missed injury later [11,23]. Higher ISS and hospital length of stay were not significantly associated with missed injuries in our study. In previous literature, higher ISS and longer hospital length of stay were significantly associated with a missed injury during hospital admission [10,11]. RTS and ICU length of stay are borderline. High RTS indicates a high chance of missed injury. Additionally, there was a larger likelihood of missed injury with longer ICU stays. In Giannakopoulos et al., the missed injury population had a significantly higher median length of ICU stay of nine (3.7-16.2) versus five (2-11) days [11]. Podolnick JD et al. had shown that the median ICU length of stay for pediatric patients with a missed injury was two days versus one day for patients without a missed injury [27].

Limitations

One of the limitations of this study was that it was performed in a single center; therefore, it may not be representative of the general population. A comparison of outcomes in other hospitals can offer more accurate reporting. Additionally, the results could be underestimated, as some patients may seek further treatment in another hospital after discharge and heal without clinical reassessment and follow-up in KAMC.

## Conclusions

In our study, the prevalence of missed injuries was on the lower end of the spectrum in comparison to multiple published data. The most common missed injuries were fractures and joint dislocations. None of those missed injuries were life-threatening injuries or contributed to overall mortality. Higher GCS was a protective factor against missed injuries while high RTS and ICU length of stay were related to a greater likelihood of developing missed injuries during the hospital course. Thus, following the ATLS protocol to detect life-threatening injuries in trauma patients was successful; however, we recommend adhering to standardized protocols by detailed secondary and tertiary surveys, as it remains a crucial step in detecting missed injuries.
